# Assessment of Overlap of Phylogenetic Transmission Clusters and Communities in Simple Sexual Contact Networks: Applications to HIV-1

**DOI:** 10.1371/journal.pone.0148459

**Published:** 2016-02-10

**Authors:** Luc Villandre, David A. Stephens, Aurelie Labbe, Huldrych F. Günthard, Roger Kouyos, Tanja Stadler, The Swiss HIV Cohort Study

**Affiliations:** 1 Department of Epidemiology, Biostatistics, and Occupational Health, McGill University, Montréal, Québec, Canada; 2 Department of Mathematics and Statistics, McGill University, Montréal, Québec, Canada; 3 Department of Psychiatry, Douglas Mental Health University Institute, Montréal, Québec, Canada; 4 Division of Infectious Diseases and Hospital Epidemiology, University Hospital Zurich, Zurich, Kanton Zurich, Switzerland; 5 Institute of Medical Virology, University of Zurich, Zurich, Switzerland; 6 Department of Biosystems Science and Engineering, ETH Zürich, Basel, Basel-Landschaft, Switzerland; 7 Swiss Institute of Bioinformatics, Lausanne, Switzerland; IFIMAR, UNMdP-CONICET, ARGENTINA

## Abstract

**Background:**

Transmission patterns of sexually-transmitted infections (STIs) could relate to the structure of the underlying sexual contact network, whose features are therefore of interest to clinicians. Conventionally, we represent sexual contacts in a population with a graph, that can reveal the existence of communities. Phylogenetic methods help infer the history of an epidemic and incidentally, may help detecting communities. In particular, phylogenetic analyses of HIV-1 epidemics among men who have sex with men (MSM) have revealed the existence of large transmission clusters, possibly resulting from within-community transmissions. Past studies have explored the association between contact networks and phylogenies, including transmission clusters, producing conflicting conclusions about whether network features significantly affect observed transmission history. As far as we know however, none of them thoroughly investigated the role of communities, defined with respect to the network graph, in the observation of clusters.

**Methods:**

The present study investigates, through simulations, community detection from phylogenies. We simulate a large number of epidemics over both unweighted and weighted, undirected random interconnected-islands networks, with islands corresponding to communities. We use weighting to modulate distance between islands. We translate each epidemic into a phylogeny, that lets us partition our samples of infected subjects into transmission clusters, based on several common definitions from the literature. We measure similarity between subjects’ island membership indices and transmission cluster membership indices with the adjusted Rand index.

**Results and Conclusion:**

Analyses reveal modest mean correspondence between communities in graphs and phylogenetic transmission clusters. We conclude that common methods often have limited success in detecting contact network communities from phylogenies. The rarely-fulfilled requirement that network communities correspond to clades in the phylogeny is their main drawback. Understanding the link between transmission clusters and communities in sexual contact networks could help inform policymaking to curb HIV incidence in MSMs.

## Introduction

### Background and objectives

Basic epidemiologic models rest on the *random mixing* assumption [1, 2]. In the presence of random mixing, each individual in a population has a small and equal probability of coming into contact with any other individual, which can lead to very quick epidemic spread. For sexually-transmitted infections (STIs) however, the random mixing hypothesis fails to hold: STIs spread within sexual contact networks, that limit their propagation. In particular, the random mixing assumption seems ill-suited for modelling HIV-1 epidemics [2].

We conventionally represent a sexual contact network with a graph whose *nodes*, also called *vertices*, correspond to individuals, and edges, to a sexual association between them. Connected nodes are called *neighbours*. The number of neighbours for a given node is called its *degree*, and the *degree distribution* is one of the defining features of contact network graphs. The sexual contact network therefore maps potential transmission paths for sexually-transmitted pathogens. A graph may also be characterized by *community structure*, that is, it may contain distinctive, non-overlapping sets of nodes within which we observe a high connection density [3]. We call these sets *communities*.

Communities in sexual contact networks could very well leave a footprint in the observed epidemiologic history. On one hand, we expect quick (early) transmission within a recently-infected community. Indeed, right after a virus infects a first node in a community, the number of edges leading to uninfected nodes is high, which decreases the mean time until the next infection event. In other words, when the virus enters a new community, the number of *exposed* subjects, that is, uninfected neighbours of infected subjects, tends to rise quickly, and the incidence is expected to spike as a result. On the other hand, we expect slow (late) transmission between different communities [4]. After all, in a contact network sense, communities have to be distinctive, which implies that their member nodes tend to be considerably more frequently connected with one another than with non-member nodes.

Our study explores the association between communities and epidemic spread, the transmission history being represented with a bifurcating tree known as a *phylogeny*. *Phylogenetic methods* make use of sequencing data to reconstruct the ancestral history of a set of organisms, such as HIV-1, and it is still unclear to what extent community structure can be recovered from the phylogeny.

### A formal definition of community structure

There are two requirements for a set of nodes to be called a community: a large enough proportion of nodes in the set must be connected to one another, and the set must be sparsely connected to external nodes [5]. Formally, a proposed split of nodes into communities is evaluated using a *modularity* score [3]. Different node partitions are proposed and scored, the objective being to identify the partition that maximizes this score: a higher maximum modularity implies a more apparent community structure.

Computing the modularity of all possible node partitions quickly becomes intractable as network size increases and so, communities in graphs are usually found heuristically. For example, the *walktrap* and *label propagation* algorithms are two basic community-detection algorithms [6, 7]. Several more sophisticated approaches can also be used [5]. In large, complicated graphs, different algorithms may yield vastly different results, whose validity can be assessed based on substantive criteria such as a priori knowledge of the relatedness of subjects in the network, if this information is available, or through the modularity score.

**Fig 1:**
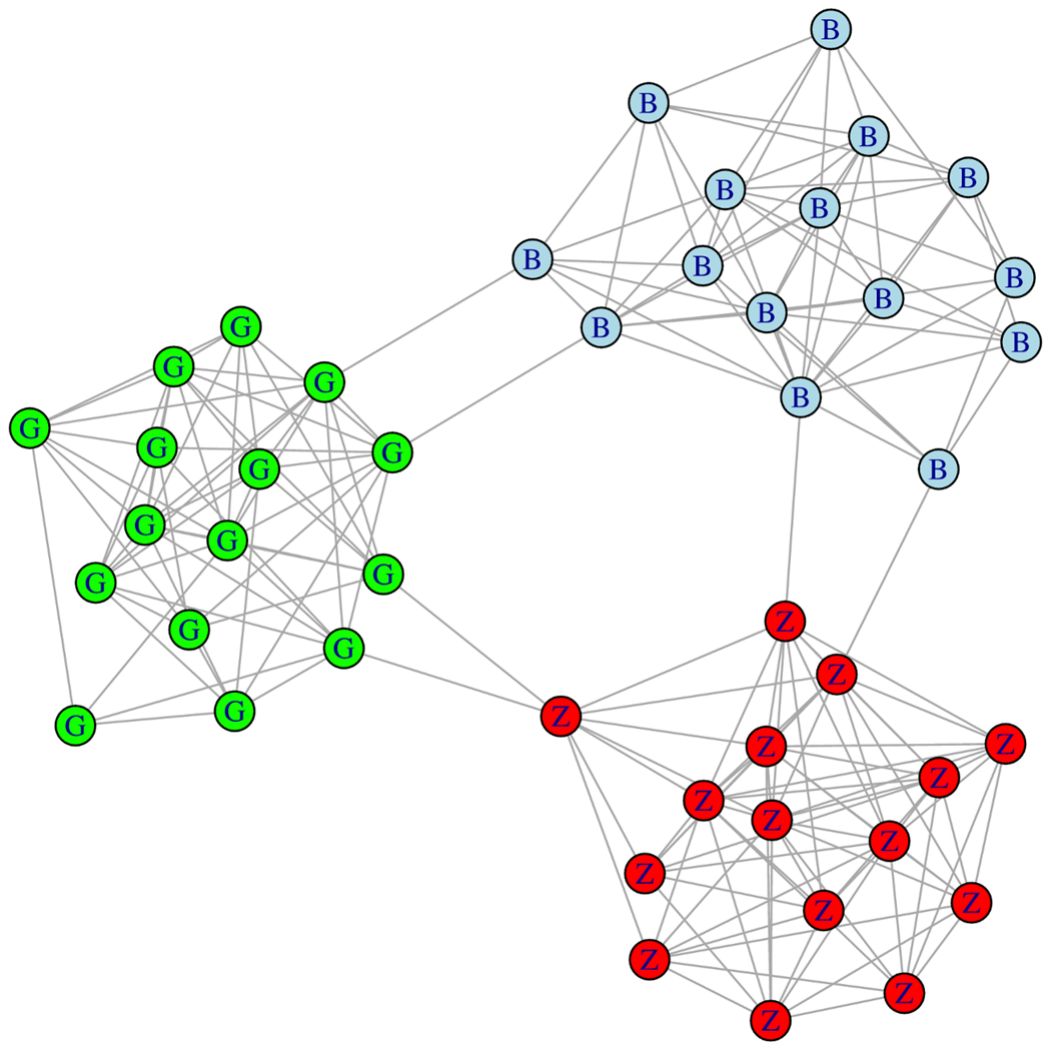
A simple undirected interconnected-islands network, representing subjects living in three different islands, corresponding to cities. The graph was randomly-generated. It has three islands of of size 15, and the connection probability of any two vertices within an island is 0.6. There are two edges, called “bridges”, between any two islands. The label within each vertex indicates in which city the subject lives.

In *undirected interconnected-islands models* however, characterized by disjoint subnetworks connected by bridges (see Fig 1), it is straightforward to make all algorithms return the optimal partition: in these models, each island corresponds to a community. Although simplistic, such networks are not entirely unrealistic. Islands could be thought of as subnetworks in separate countries, cities, or neighbourhoods. They can also result from a high degree of *assortative mixing*. Assortative mixing refers to the tendency of subjects to form connections with individuals sharing similar characteristics, for example, age, socio-economic status, or profession. A high degree of assortative mixing tends to produce easily-distinguishable regions in the contact network graph, made up of nodes representing similar subjects. More specifically, sociodemographic characteristics such as age could contribute to producing islands within a localized sexual contact network.

### The link between contact networks and phylogenies

Several studies have looked at the link between contact networks and phylogenies, producing seemingly mixed conclusions. [8] investigated how *clustering* in a contact network graph, defined as the propensity of pairs of connected nodes to share a common neighbour, affects epidemics, in terms of their phylogenies. They found that changes in network clustering result in very little variation in trees. [2], on the other hand, found a strong association between the shape of phylogenies resulting from epidemics on four types of static contact networks. [9] extended the investigation of [2] by, among other things, looking at a family of dynamic networks. Unlike the latter, they found only a modest direct effect of network configuration on phylogenies. Another study presents an attempt to directly reconstruct a sexual contact network, underlying HIV transmission, based on epidemiological and genetic information [10].

### The relevance of sexual contact networks: HIV-1 epidemics among men who have sex with men

HIV-1 remains at a high prevalence among men who have sex with men (MSMs) in developed countries. In the United States, in 2010, MSMs represented 63% of the estimated total number of new HIV infections, and the prevalence of HIV in this subpopulation was approximately 18% [11]. Despite an increase in the proportion of individuals treated with *highly active antiretroviral therapy* (HAART), the incidence rate of HIV in MSMs in the United States has increased by an estimated 12% between 2008 and 2010.

#### Quick transmission chains

Studies have revealed the existence of *quick transmission chains* in HIV epidemics among MSMs [12, 13], that is, distinct groups of infected subjects with genetically-similar viruses. Such groups can only be formed through series of infection events close in time. Indeed, the fast evolution of HIV-1 ensures that correspondence in the genetic makeup of viruses in different subjects indicates not only epidemiologic relatedness, but also a recent viral common ancestor. In other words, sets of genetically similar infections must result from chains of quickly-occuring transmissions. We stress that a quick transmission chain does not necessarily involve a first individual transmitting the virus to a second individual, who then infects a third one, and so on. We can also have a single infected subject transmitting the virus to an arbitrary number of individuals in a short amount of time. The “chain” is defined with respect to transmission events, irrespective of the transmission path.

Understanding the reasons for the existence of quick transmission chains is crucial, as it could inform public health interventions in MSMs. For instance, the WHO now advocates Treatment as Prevention (TasP) [14]. However, the potential of TasP to curb the HIV-1 epidemic in this subpopulation depends in great part on the timing of onward transmission. If it tends to occur mostly within the first month after infection, corresponding to the *acute infection stage* [15], TasP is unlikely to succeed in significantly reducing incidence rates, since HIV-1 is rarely diagnosed so early [16]. Indeed, the detection of numerous quick transmission chains may suggest that early transmissions are mainly responsible for the rising HIV-1 incidence rates in MSMs [13]. In this context, TasP could still potentially prevent a number of infections, but would be unsuccessful in containing the epidemic.

#### Transmission clusters

Quick transmission chains for HIV correspond to *transmission clusters*, loosely defined as sets of HIV-positive individuals whose viruses share a “close” common genetic ancestor [17]. How close this common ancestor ought to be is typically decided in an ad hoc way. Sets of co-clustered subjects have viruses that are not only close in genetic terms, but also distinct from any viruses from non-co-clustered subjects.

Numerous studies have used phylogenetics for transmission cluster inference, and have reported the existence of large transmission clusters in HIV epidemics among MSMs [13, 18–20]. In MSMs, transmission clusters may explain as much as 75% of incidence, with one infection leading up to an estimated 10 to 13 onward transmissions [4, 17].

#### Sexual contact networks in MSMs

The existence of quick transmission chains should inform public health approaches and so, gaining insight into the factors contributing to time between transmissions is important. One such factor could be the structure of the sexual contact network: it might play a major role in the production of quick transmission chains.

Sexual contact networks in MSMs are characterized by high clustering, assortative mixing, and the presence of several nodes with a distinctively larger degree [21]. Clustering, in a contact network graph, refers to the frequency at which any pair of nodes share a common neighbour. The existence of several distinctive high-degree nodes may result from *preferential attachment*, which implies that each subject, when forming a new connection, tends to prefer individuals with an already large number of connections. Preferential attachment leads to graphs with several “hubs”, nodes with an exceptionally high number of neighbours. The resulting degree distribution, called a *power-law distribution*, therefore has a heavy right tail. Communities may result from a combination of assortative mixing and preferential attachment.

#### Understanding communities for prevention of HIV-1 in MSMs

The existence of numerous transmission clusters, because of their correspondence with quick transmission chains, may explain the difficulties in containing HIV-1 incidence in MSMs [17], but its link with community structure in MSM sexual contact networks is poorly understood. Assessing the contribution of community structure to HIV-1 incidence in MSMs may be helpful to design more effective intervention strategies. With this goal in mind, our study looks specifically at how well transmission clusters map onto communities in a graph. In other words, findings in the present study will help understand the extent to which HIV-positive MSMs in the same transmission cluster tend to belong to the same community, defined with respect to a graph, within a known network structure with easily-distinguishable communities.

## Methods

Since we do not know of any extensively mapped sexual contact network for MSMs and infection tracing is difficult in this population [22], we have no choice but to rely on simulations.

### Simulating the sexual contact networks

In each simulation, we simulate a sexual contact network, and then, an epidemic spreading onto it. We simulate epidemics on three classes of randomly-generated networks, all within the framework of undirected interconnected-islands models: two deliberately simplistic, called “type” A and “type B”, and the other, called “type C”, tailored in such a way that it displays prominent features of real sexual contact networks. Weights in our networks modulate distance between any two connected subjects. In unweighted networks, any two connected subjects are at arbitrary distance 1. In weighted networks, we attribute weights below 1 to edges serving as bridges. Distance between any two subjects is equal to one over the sum of weight values for edges on the shortest path between them. It follows that the two subjects delimiting a bridge are more distant when we attribute it a lower weight. In each weighted network, we give all bridges the same weight.

#### Network consisting of many islands of equal size (Type A)

In the first network structure, we find 13 islands of 20 subjects each, with each island being a *fully-connected graph*, and one bridge linking any two islands. In a fully-connected graph, all vertices are connected to each other. We let bridge weights take values 0.25, 0.5, 0.75, or 1. In the latter case, the network is unweighted.

#### Network consisting of one large island connected to many small islands (Type B)

The second network structure consists of one central island of size 60, representing a foreign sexual contact network, connected by single bridges to 25 islands of size 20. Each small island is a fully-connected graph. We implement weighting exactly as before. The set of small islands represents disjoint sexual contact subnetworks in a population of interest.

#### The more realistic network (Type C)

The more realistic networks are made of 100 islands each. To ensure that all islands are accessible, we first link islands in a chain. We then create additional bridges by connecting any two vertices belonging to different islands with probability 0.00075. As in networks of types A and B, we consider networks in which bridge weights take values 0.25, 0.5, 0.75, or 1.

We introduce preferential attachment by making each island a *Barabasi-Albert* graph [23]. In our simulations, each subnetwork is generated by first creating three interconnected subjects. New subjects are then added one by one. When introduced into the network, a subject randomly forms three connections with existing subjects, who become first-degree neighbours. The probability that a subject is selected increases linearly with his degree. We call this *linear preferential attachment*.

Further, islands in these networks are of variable size. We sample island sizes independently from an empirical distribution obtained by inspection of the maximum likelihood phylogeny for HIV-1 subtype B sequences used in [24]. All viral sequences come from participants in the Swiss HIV Cohort Study (SHCS). We use this phylogeny to partition the 5395 sampled HIV-positive subjects into transmission clusters. We consider co-clustered sets of subjects whose viral sequences are separated by a tree distance of at most 0.1 nt/bp, forming a *clade* of size 5 or more, with this clade having bootstrap support greater than 70%. A set of tips forms a clade if and only if there is a node in the tree with its descending tips corresponding exactly to this set of tips. Based on these criteria, we obtain a list of clusters, for which we derive a cluster size distribution. This distribution may have gaps and so, we apply a loess smoother to it [25].

Because [24] are working with empirical HIV sequencing data, many HIV-positive subjects who would co-cluster with individuals in the sample may be missing. This incomplete sampling may result from undiagnosed subjects or diagnosed subjects that could or would not participate in the SHCS [20]. In order to account for this, we assume that each known infection is connected to either 0, 1, or 2 unknown infections, with an average of 1 unknown infection.

### The link between islands and communities

In our analyses, we assume that each island is a community. We ensure that two conventional community-detection algorithms validate this assumption. That is why we measure, with the adjusted Rand index (ARI) [26], correspondence between subjects’ island and community membership indices, with the latter indices produced by the walktrap and label-propagation community-detection algorithms [6, 7]. The ARI is equal to the ratio of correctly co-clustered and separated elements to the total number of pairs of elements, with a correction term for chance. It is bounded above by 1, which in our case indicates perfect overlap between islands and communities. It gives insight similar to the adjusted mutual information score.

### Simulating the epidemics

Subjects can be in one of three states: “susceptible”, “infected”, or “removed”. All subjects start in the “susceptible” state. After introduction of the virus in the network, susceptible subjects may contract the virus from an infected neighbour, at which point they enter the “infected” state. Infections are eventually diagnosed, and subjects move to the “removed” state. In the context of HIV, removal corresponds to diagnosis and uptake of HAART, which prevents transmission by drastically reducing the viral load.

We stop simulating new transmissions once a predetermined number of subjects are in the “removed” state or, trivially, once the epidemic becomes *extinct*, that is, all infected subjects have moved on to the “removed” state.

We represent each simulated epidemic with a transmission tree connecting the infected individuals. We terminate a lineage, that is, we obtain a tip, upon an individual leaving the “infected” state and entering the “removed” state. In the analyses, we consider only the subtree connecting all tips corresponding to removed individuals. This phylogeny lets us partition those subjects into transmission clusters. This scheme reflects real data collection, in which an infection can only be recorded after diagnosis.

In type A sexual contact networks, epidemics result from one random introduction of the virus. In type B networks, we randomly introduce the virus once in the large island, but we assume that only subjects in the small islands can be observed. It follows that removed subjects in the large island are not counted for the purpose of determining when to stop the simulations, as they belong to a foreign epidemic. Thus, the tips in the phylogenies representing the simulated epidemics only correspond to removed individuals in the small islands. Given the large size of the type C networks, we increase the number of introductions to two. This increase also aims to produce a more plausible epidemic, as multiple introductions are common in practice [20].

Transmission time along any edge follows an exponential distribution with rate directly proportional to the associated weight, or equivalently, inversely proportional to the distance between the subjects delimiting the edge. In type A and type B networks, we stop simulating new infections once 60 subjects have entered the “removed” state. In type C sexual contact networks, because of their large size, we raise this requirement to 200 subjects. If an epidemic fails to reach this threshold, we discard it along with the sexual contact network on which it was simulated. We then generate a new network and a new epidemic. We simulate epidemics until 300 manage to reach the threshold.

The minimal size requirement ensures that all sampled epidemics are of the same size and span more than one community, which makes comparisons across and within scenarios more straightforward. Indeed, since an increase in the distance between islands reduces mean time to extinction, without the size requirement, scenarios with more distant islands would involve systematically smaller epidemics, with more of them becoming extinct before a single bridge is crossed. In that extreme case, the ARI for community recovery would be trivially 0, which could be misleading. Indeed, the ARI is meaningful only when the data are partitioned in a non-trivial way. Further, averaging ARIs obtained on epidemics of different sizes would be questionable, since epidemic size affects the difficulty of the clustering problem.

In order to reduce the probability of epidemics going extinct too early, we use a shifted exponential distribution to generate time until removal. In other words, this time corresponds to the sum of a fixed time and a value simulated from an exponential distribution. It follows that the distribution of time until removal is bounded below by a value greater than zero. The shift gives all newly-infected subjects a minimum amount of time to potentially transmit the virus to their susceptible neighbours. A subject enters the “removed” state at the moment of diagnosis and so, this delay is understood as reflecting the time it takes for an infection to become diagnosable.

Time in the simulation of epidemics corresponds to genetic distance. In other words, it is understood as the expected number of mutations divided by the number of loci in the DNA alignment. Under a strict molecular clock assumption, genetic distance translates directly to calendar time. We represent each simulated epidemic by a rooted phylogeny, with which, if needed, we can obtain simulated samples of DNA sequences. To get such a sample, first, we simulate a *root sequence*, that is, the genetic makeup of the virus that first entered the sexual contact network, assuming equal frequency for all four bases. Evolution along the phylogeny follows a continuous time Markov process, whose rate matrix we inferred from a subsample of HIV-1 subtype B sequences collected in Montreal, Québec, Canada. By letting the root sequence evolve along the phylogeny, we obtain the required sample.

### Finding the clusters and assessing correspondence to islands

There is no widely-accepted method for partitioning samples of sequencing data into transmission clusters, and different methods tend to yield contradicting results. To ensure that our analyses are not overly affected by the arbitrary selection of one method, we obtain transmission clusters in four different ways, all commonly employed in HIV transmission cluster inference. We define a transmission cluster as,

1. A clade whose elements are separated by a fixed tree distance of at most *x*, where tree distance is the sum of branch lengths between any two tips [12],
2. A clade whose elements are separated by a fixed distance of at most *x*, where distance is the standardized number of different nucleotides between any two sequences [27],
3. A clade whose elements are separated by a median pairwise tree distance below *x*, an arbitrary percentile of the tree’s between-tip tree distance distribution [28], where tree distance once again corresponds to the sum of branch lengths,
4. A clade in a dendrogram, that is, an ultrametric tree obtained from the matrix of between-tip tree distances, cut at height *x*, where the dendrogram is obtained using one of three methods: average linkage, complete linkage, or *Weighted Pair-Group Method of Analysis* (WPGMA),

where we let *x* vary in order to inspect its effect on community recovery.

Methods in definition 4 are standards in agglomerative hierarchical clustering, which involves recursively combining the two clusters that are closest one to another [29]. The three selected methods differ in how they define inter-cluster distance. In the average linkage method, distance between two clusters is an average of genetic distance within all non-co-clustered pairs of elements from these clusters. In the complete linkage method, this distance is instead the maximum distance within those pairs. In WPGMA, distance between two clusters is the distance between two elements, one in each cluster, each deemed the most representative of its cluster.

Under definitions 1, 2, and 3, the *clade-based methods*, we explore the tree from root to tips to verify if nested clades meet the clustering requirements. More specifically, we employ a *depth-first* algorithm,

1. Start exploration at the root node,
2. Check if the clade whose *most recent common ancestor* (MRCA) is the current node meets the cluster definition. If so, stop. If not, move down to the two immediate children nodes and for each of them, repeat the current step,
3. Stop exploration along any given path once a tip is reached.

In real studies, transmission cluster inference additionally relies on a confidence requirement for clades in the phylogeny. In our analyses however, we use the true phylogeny and so, all clades are known with confidence 1. We assume that each island is a community and we measure overlap between transmission clusters and islands with the adjusted Rand index (ARI) [26].

### Ethics

Our study is a pure simulation study and so, did not require approval by an ethics committee. We used trees inferred in a study by Avila et al. [24] to obtain a component of our simulation algorithm. The latter study was conducted within the framework of the Swiss HIV Cohort Study (SHCS). The SHCS has been approved by the following ethics committees: Ethikkommission beider Basel (EKBB), Kantonale Ethikkommission Bern (KEK), Comité départemental d’éthique des spécialités médicales et de médecine communautaire et de premier recours des Hôpitaux Universitaires de Genève, Commission cantonale d’éthique de la recherche sur l’être humain du canton de Vaud, Comitato etico cantonale—Cantone Ticino, Ethikkommission des Kantons St.Gallen, Kantonale Ethik-Komission Zürich (KEK). The data collection was anonymous and written informed consent was obtained from all participants. The detailed ethics approval for the SHCS can be found at http://www.shcs.ch/206-ethic-committee-approval-and-informed-consent.

### Software

We perform simulations and cluster inference in R v3.1.2, using functions contained in the *igraph*, *ape*, and *phangorn* packages [30, 31]. We validate results obtained for cluster definitions 2 and 3 by comparing them to those from ClusterPicker v1.3 and PhyloPart v2.0, respectively. Code is available upon request.

## Results

### Networks and phylogenies

In order to highlight the association between phylogenetic and network representations of epidemics, we plot two toy examples, involving simulated epidemics on unweighted and weighted networks of type A.

**Fig 2:**
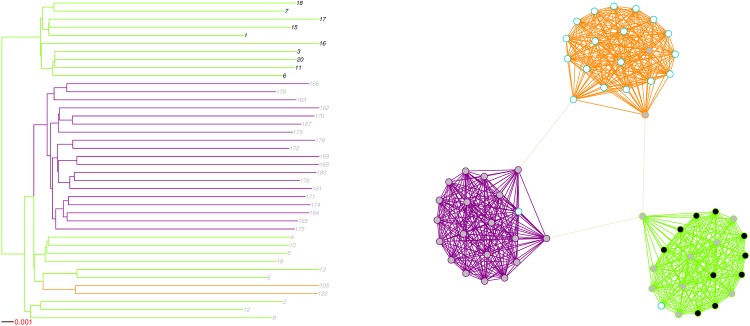
Phylogeny and associated unweighted network graph for a simulated epidemic. Branch colour indicates on which island transmission and evolution takes place, while tip label colour indicates cluster membership based on snipping the WPGMA dendrogram at height 0.007. Only islands with at least one infected vertex are displayed. Vertex and edge colours are matched with tip label and branch colours, respectively. A white vertex with a teal frame is infected, but undiagnosed. A white vertex with frame color matching that of the island is uninfected.

We show a simulated network on the right in Fig 2. Each island is identified by a colour: purple, orange, and green. The virus was introduced in the green island and then travelled to the purple island. A subject in the green island was also responsible for transmitting the virus to the orange island. On the left in Fig 2, we have the epidemic’s phylogenetic representation. Branch colour indicates on which island sequences are evolving and transmissions are taking place. Tip label colours indicate to which transmission cluster, black or grey, a given sequence belongs and are matched with node colours in the network graph. We obtained those clusters by applying the WPGMA method (Def. 4) and selecting the cutpoint that maximized correspondence between islands and clusters. The network graph emphasizes the limited correspondence between the transmission clusters we obtained and the islands. Indeed, subjects in the grey cluster were found on all three islands and shared the green island with the 10 subjects in the black cluster.

The plotted phylogeny clearly emphasizes that any clustering method assuming that a cluster is a clade cannot achieve high island recovery, as infected individuals from the same island may not form a clade. Indeed, in our example, infected individuals in the orange and the purple islands formed a clade in the phylogeny, while infected individuals in the green island did not. In general, individuals from an island with ongoing transmission do not induce a clade if one of these individuals transmits to a new island.

**Fig 3:**
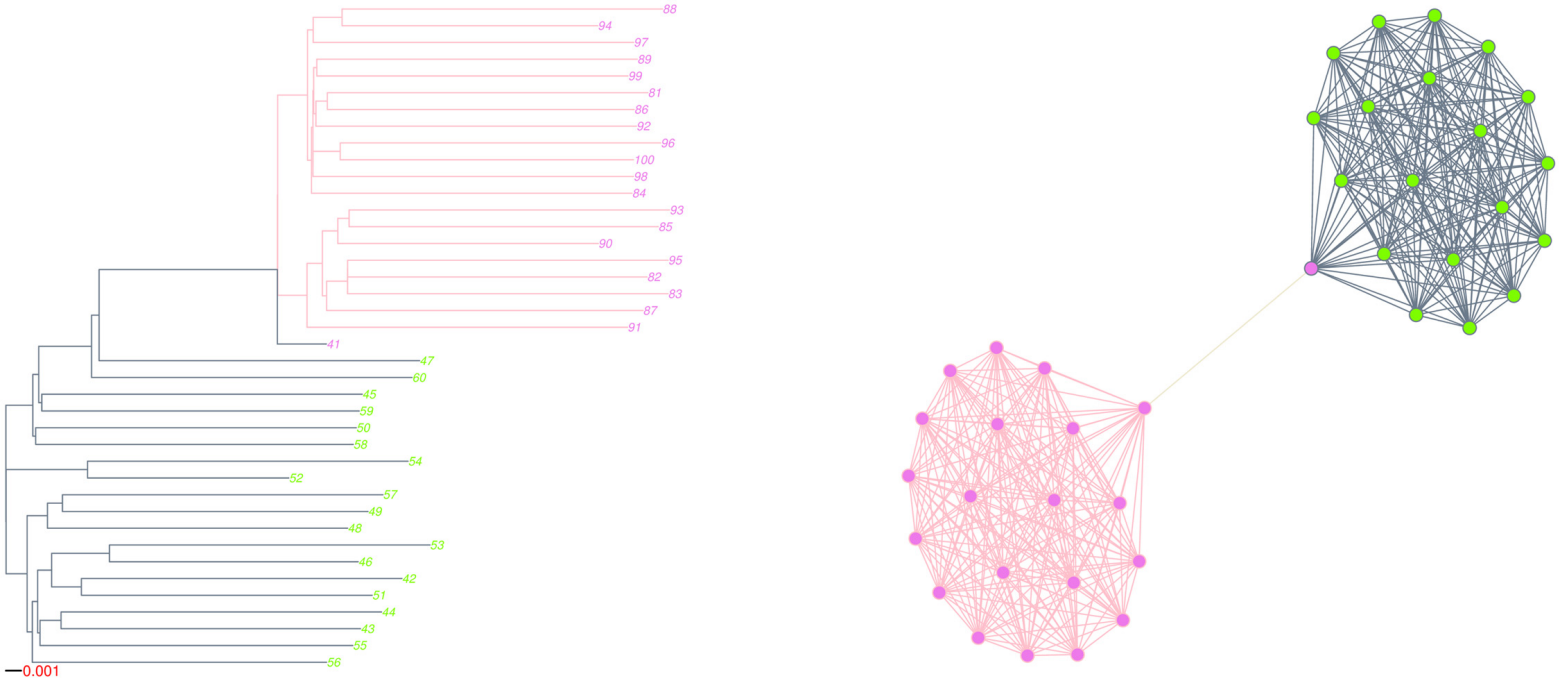
Phylogeny and associated weighted network graph for a simulated epidemic. Branch colour indicates on which island transmission and evolution takes place, while tip label colour indicates cluster membership based on snipping the WPGMA dendrogram at height 0.0071. Only islands with at least one infected vertex are displayed. Vertex and edge colours are matched with tip label and branch colours, respectively. A white vertex with a teal frame is infected, but undiagnosed. A white vertex with frame color matching that of the island is uninfected.

A visual inspection of the phylogeny can however reveal the existence of community structure, as emphasized by Fig 3, which shows an epidemic simulated on a weighted network of type A. We attributed a weight of 0.25 to bridges, thus multiplying by 4 the mean time required for the virus to cross to another island. Both islands ended up saturated. We can easily identify the branch in the phylogeny supporting the subtree representing the sub-epidemic in the pink island. This pattern is typical: when a sufficiently large number of infection events take place after a bridge is crossed, we generally observe a subphylogeny supported by a long branch, consequence of the limited number of between-island, compared to within-island, connections.

Each island was a fully-connected graph. In epidemics on fully-connected graphs, the incidence rate is proportional to the number of edges connecting infected subjects to their susceptible neighbours. Per lineage however, the transmission rate decreases with the number of infected individuals. In a phylogeny, this translates to short branches right after a crossing event, and then an increase in branch lengths.

### Epidemics in type A networks

In type A sexual contact networks, correspondence between islands and communities returned by the label propagation and walktrap algorithms was close to perfect, with ARIs slightly under 1. Type A networks had a clustering coefficient of 0.94, a mean degree of 19.6, and a mean shortest path length of 2.76. Epidemics on these networks had an estimated reproduction number of 3.7.

**Fig 4:**
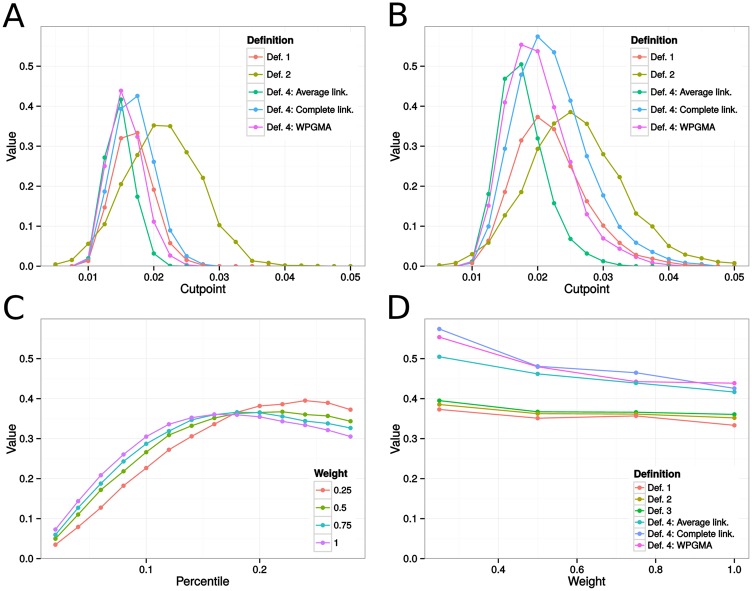
Estimates of island recovery for epidemics on type A sexual contact networks, measured with the adjusted Rand Index (ARI). Fig A indicates the mean ARI across cutpoints for the different cluster definitions applied to epidemics simulated on unweighted networks. In Fig B, we show corresponding results for weighted networks with between-island transmission rate equal to 25% the within-island transmission rate. In Fig C, each curve gives mean island recovery rates across between-tip distance percentile requirements in Def. 3 for networks with between-island transmission rates 25%, 50%, 75%, or 100% the within-island transmission rate. Fig D gives the optimal ARIs across between-island transmission rates. Each curve represents the maximum achieved mean island recovery under the different cluster definitions. For example, the values at bridge weight 0.25 are the suprema in Fig B, as well as the supremum of the red curve in Fig C. Def. 1-4 correspond to the methods for cluster detection described in the Methods section.

ARIs varied widely across simulations. For instance, at cutpoint 0.02, on networks with bridge weights set at 0.25, clusters obtained from the complete linkage method produced ARIs between 0, meaning that we concluded in the absence of clusters, to 0.88, yielding a variance of 0.02. Fig 4 gives mean island recovery rates across distance requirements under the different weighting schemes for the cluster definitions stated previously. Overall, we observed low to moderate correspondence between transmission clusters and communities, with mean recovery rates lower when epidemics spread on unweighted networks. Around the optimal cutpoint, clusters obtained under Def. 4 tended to agree more with the island structure, as evidenced by Fig 4D, with complete linkage usually producing better correspondence.

Fig 4A and 4B further suggest that complete linkage worked better under a wider range of cutpoints. However, past this optimal range, Def. 2 produced greater overlap with the islands. Fig 4C and 4D show that even under the optimal percentile threshold, Def. 3 produced clusters that overlapped modestly with the islands, although it did slightly outperform Def. 1 and Def. 2 under optimal setting.

Fig 4A and 4B emphasize how weighting affects cluster recovery. In clusters resulting from the use of hierarchical clustering with complete linkage, from above 0.58 when bridge weights were set at 0.25, the ARI decreased to approximately 0.43 in the unweighted network case. Weighting also improved optimal recovery rates for Def. 1, 2, and 3, although the variation was much smaller.

### Epidemics in type B networks

The mean ARIs for correspondence between the small islands in type B sexual contact networks and communities returned by the walktrap and label propagation algorithms were also very close to 1. Type B networks had a mean clustering coefficient of 0.98, a mean degree of 17.7, and a mean shortest path length of 5.9. Epidemics on these networks also had an estimated reproduction number of 3.7.

**Fig 5:**
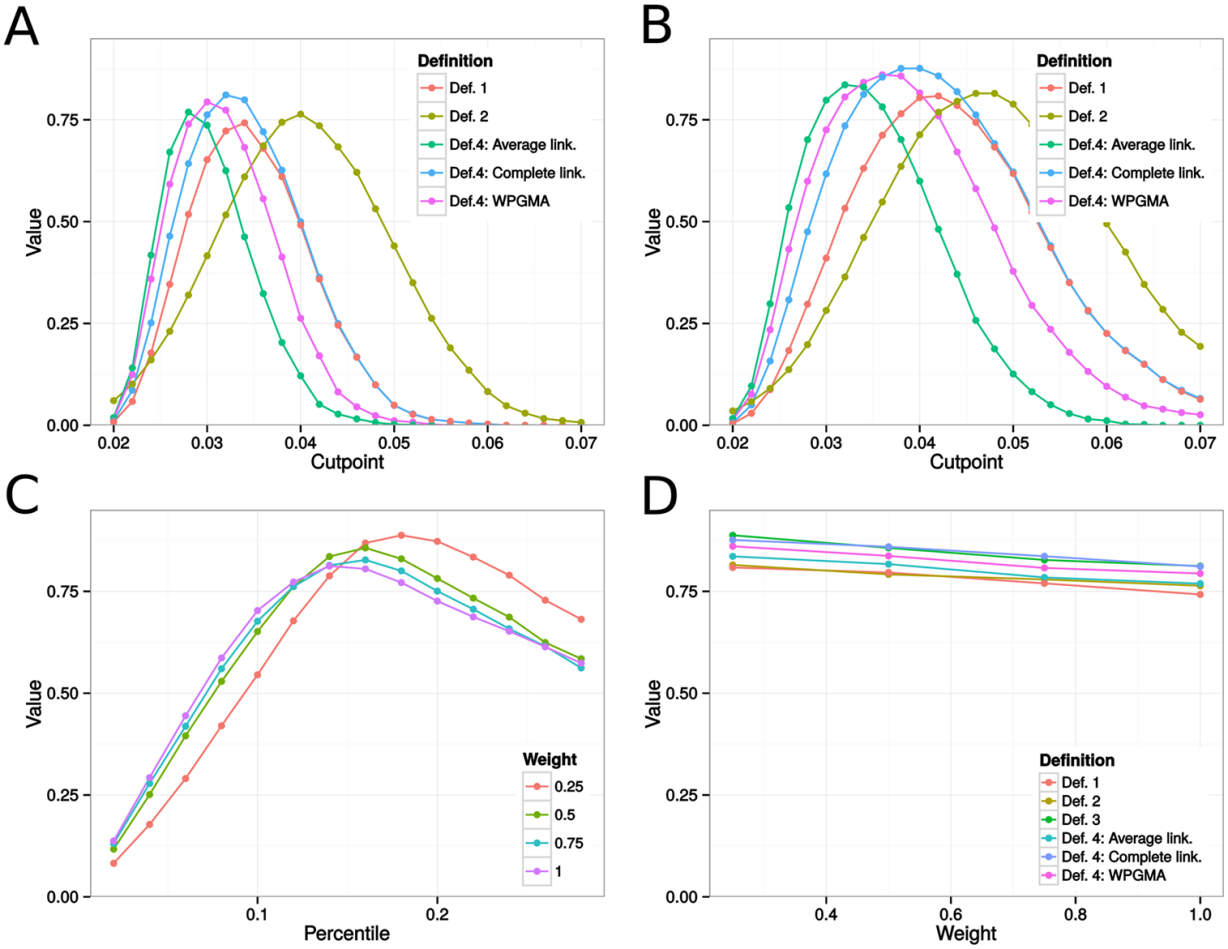
Estimates of island recovery for epidemics on type B sexual contact networks, measured with the adjusted Rand Index (ARI). Fig A indicates the mean ARI across cutpoints for the different cluster definitions applied to epidemics simulated on unweighted networks. In Fig B, we show corresponding results for weighted networks with between-island transmission rate equal to 25% the within-island transmission rate. In Fig C, each curve gives mean island recovery rates across between-tip distance percentile requirements in Def. 3 for networks with between-island transmission rates 25%, 50%, 75%, or 100% the within-island transmission rate. Fig D gives the optimal ARIs across between-island transmission rates. Each curve represents the maximum achieved mean island recovery under the different cluster definitions. For example, the values at bridge weight 0.25 are the suprema in Fig B, as well as the supremum of the red curve in Fig C. Def. 1-4 correspond to the methods for cluster detection described in the Methods section.

As before, we observed large variations in ARIs across simulations. For example, on networks with bridge weights taking value 0.25, clusters obtained using the complete linkage method, at optimal cutpoint 0.04, had ARIs ranging from 0.02 to 1, producing a variance of approximately 0.02. Fig 5 shows that all cluster definitions could be tuned to recover the island structure fairly accurately. In the weighted case (Fig 5B), optimal island recovery reached over 0.90. Once again, hierarchical clustering (Def. 4) tended to perform better, but the difference in optimal recovery rates was rather small. Like before, Def. 2 clearly outperformed other definitions at higher cutpoints. The improved performance of clade-based definitions was not a surprise. After all, in type B networks, all epidemic outbreaks in the smaller islands form clades in the phylogeny.

### Epidemics in type C networks

The more realistic networks were of mean size 985.70, with mean degree 5.70, mean clustering coefficient 0.37, and mean shortest path length 5.64. Islands had sizes ranging from 5 to 16, with mean 9.84. Approximately 9.63% of islands were of size 5. The island size distribution frequency decreased slowly, but systematically, with increasing size until it reached 2.90% for islands of size 16 (S1 Fig). Within-island degree distributions had the heavy right tail resulting from preferential attachment (S2 Fig). Communities returned by the label propagation algorithm overlapped strongly with the islands, with an ARI close to 0.99. With an ARI of 0.80, the communities proposed by the walktrap algorithm still matched the islands rather closely. Epidemics on type C networks had an estimated reproduction number of 2.35.

**Fig 6:**
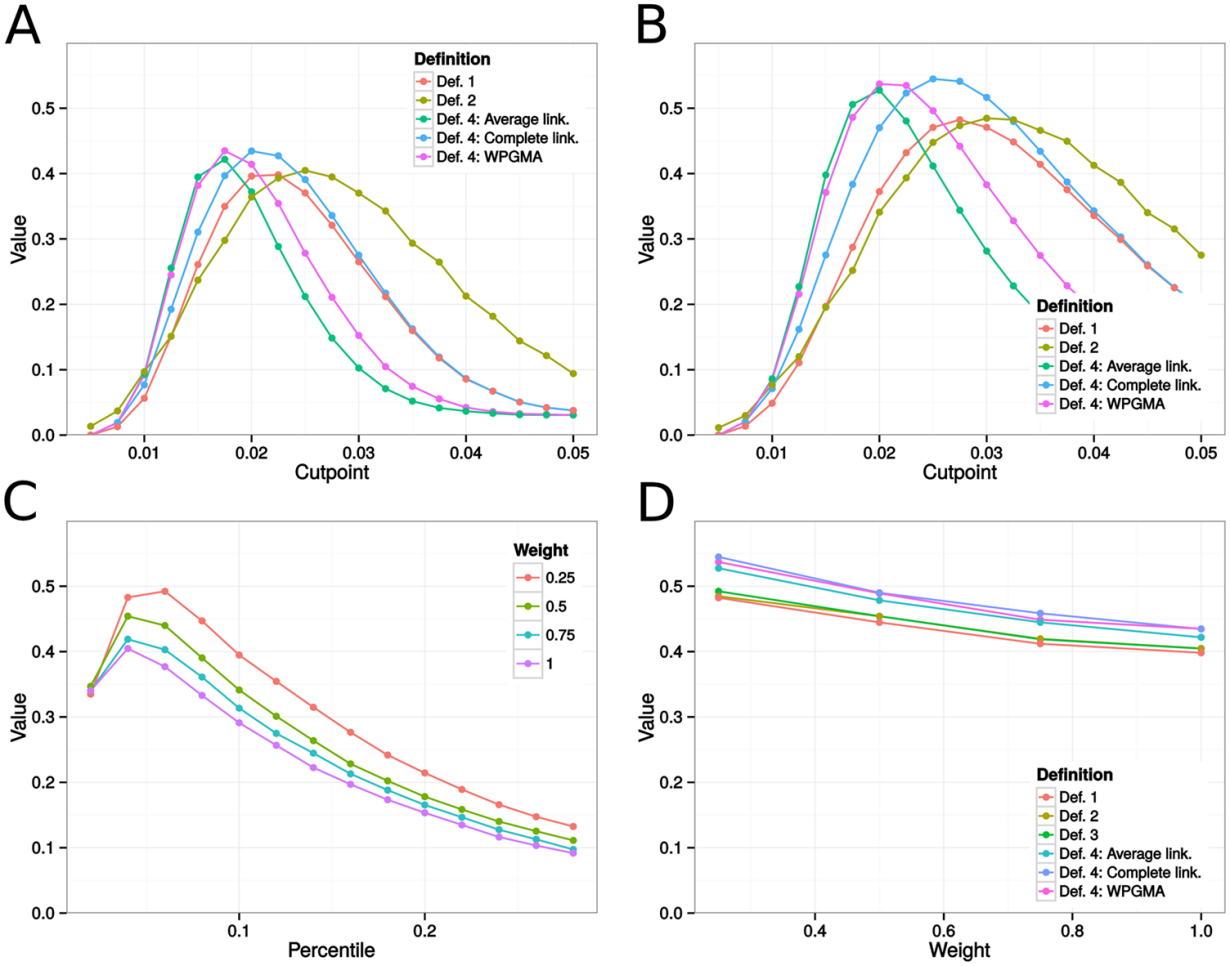
Estimates of island recovery for epidemics on type C sexual contact networks, measured with the adjusted Rand Index (ARI). Fig A indicates the mean ARI across cutpoints for the different cluster definitions applied to epidemics simulated on unweighted networks. In Fig B, we show corresponding results for weighted networks with between-island transmission rate equal to 25% the within-island transmission rate. In Fig C, each curve gives mean island recovery rates across between-tip distance percentile requirements in Def. 3 for networks with between-island transmission rates 25%, 50%, 75%, or 100% the within-island transmission rate. Fig D gives the optimal ARIs across between-island transmission rates. Each curve represents the maximum achieved mean island recovery under a different cluster definition. For example, the values at bridge weight 0.25 are the suprema in Fig B, as well as the supremum of the red curve in Fig C. Def. 1-4 correspond to the methods for cluster detection described in the Methods section.

Because of the larger size of the epidemics, variability in ARIs obtained across simulations was lower than before. For example, in networks with bridge weights set to 0.25, at optimal cutpoint 0.025, clusters inferred using the complete linkage method lead to ARIs ranging from 0.39 to 0.70, with a variance of 0.003. Fig 6 presents mean island recovery rates across distance requirements under the different weighting schemes and cluster definitions. Despite considerable differences between networks of type A and type C, the results were similar to those outlined before. Once again, hierarchical clustering algorithms (Def. 4) suggested transmission clusters that overlapped more with islands, and among those algorithms, the complete linkage method performed better, as it worked better under a wider range of cutpoints and produced slightly higher suprema.

The other three definitions offered a better performance than in networks of type A. We suggest that this is due to the increase in the number of introductions and the larger number of infected islands, leading to infected individuals from the same island forming clades in the phylogeny more often.

## Discussion

Disagreements persist about the interpretation of transmission clusters inferred from phylogenies [28]. The present work illustrates how transmission clusters obtained by applying common methods may overlap only partially with islands in the underlying network, especially when epidemics result from a small number of introductions. Because hierarchical clustering methods do not require clusters to be clades in the phylogeny, they tended to produce clusters that corresponded more closely to islands. The three clade-based methods performed almost as well as hierarchical clustering when we simulated under scenarios where each transmission chain within an island formed a clade (type B networks). Since the simulated islands form clear-cut communities, we conclude that community structure cannot be inferred reliably using the existing phylogenetic clustering tools.

The present study does have limitations. Real genotyping data from a mapped transmission network was unavailable and so, we had to rely on simulations. It follows that we cannot establish without a doubt that our results would generalize beyond the scenarios we selected. Nevertheless, the similarities between the results we obtained across network types make us confident that our conclusions are reasonably robust to the characteristics of the network structure. We recognize that the networks we simulated are major simplifications of empirical networks, but they do exhibit some important properties of real sexual contact networks, namely moderate to high clustering, community structure, and low mean shortest path lengths.

The island network model has the important advantage of comprising unambiguous communities. If transmission clusters do not approximate communities in such networks, it is unlikely they will correspond to communities in other network models.

In summary, current widely-used phylogenetic clustering methods (Def. 2 and 3, which are extensions of Def. 1) often failed to recover sexual contact network communities reliably. A major drawback was their assumption that clusters form clades in the phylogeny, while many clusters were not clades, like in the epidemics simulated in type A and type C networks. Conventional hierarchical clustering methods outperformed the more sophisticated methods in the case where clusters were not clades, and produced similar performance in networks where clusters always formed clades, like in networks of type B. However, particularly for epidemics in type A and type C networks, hierarchical clustering was still far from optimal. Therefore, we call for new clustering methods improving on existing approaches, by dropping for instance the clade assumption.

Understanding the link between HIV transmission clusters and community structure in sexual contact networks could help inform policymaking to curb HIV incidence in MSMs. This work stresses the need for new clustering algorithms that focus on community recovery, which remains limited and incidental under current methods. We speculate that covariate information, such as sociodemographic characteristics, could be helpful in community detection, and could best be integrated within a novel parameteric approach that, unlike conventional methods, would not rely on the clade assumption and ad hoc requirements.

## Supporting Information

S1 Fig Island size distribution in type C networks. (TIF)

S2 Fig Within-island degree distributions, stratified on island size, in type C networks. (TIF)

S1 File Networks of type A—Bridge weights are equal to 1. (RDATA)

S2 File Networks of type A—Bridge weights are equal to 0.75. (RDATA)

S3 File Networks of type A—Bridge weights are equal to 0.5. (RDATA)

S4 File Networks of type A—Bridge weights are equal to 0.25. (RDATA)

S5 File Networks of type B—Bridge weights are equal to 1. (RDATA)

S6 File Networks of type B—Bridge weights are equal to 0.75. (RDATA)

S7 File Networks of type B—Bridge weights are equal to 0.5. (RDATA)

S8 File Networks of type B—Bridge weights are equal to 0.25. (RDATA)

S9 File Networks of type C—Bridge weights are equal to 1. (RDATA)

S10 File Networks of type C—Bridge weights are equal to 0.75. (RDATA)

S11 File Networks of type C—Bridge weights are equal to 0.5. (RDATA)

S12 File Networks of type C—Bridge weights are equal to 0.25. (RDATA)
