# Supplementary figures and images for: Assessment of Overlap of Phylogenetic Transmission Clusters and Communities in Simple Sexual Contact Networks: Applications to HIV-1
Source: PLoS One. 2016 Feb 10;11(2):e0148459. doi: 10.1371/journal.pone.0148459 (PMC4749335; doi:10.1371/journal.pone.0148459)

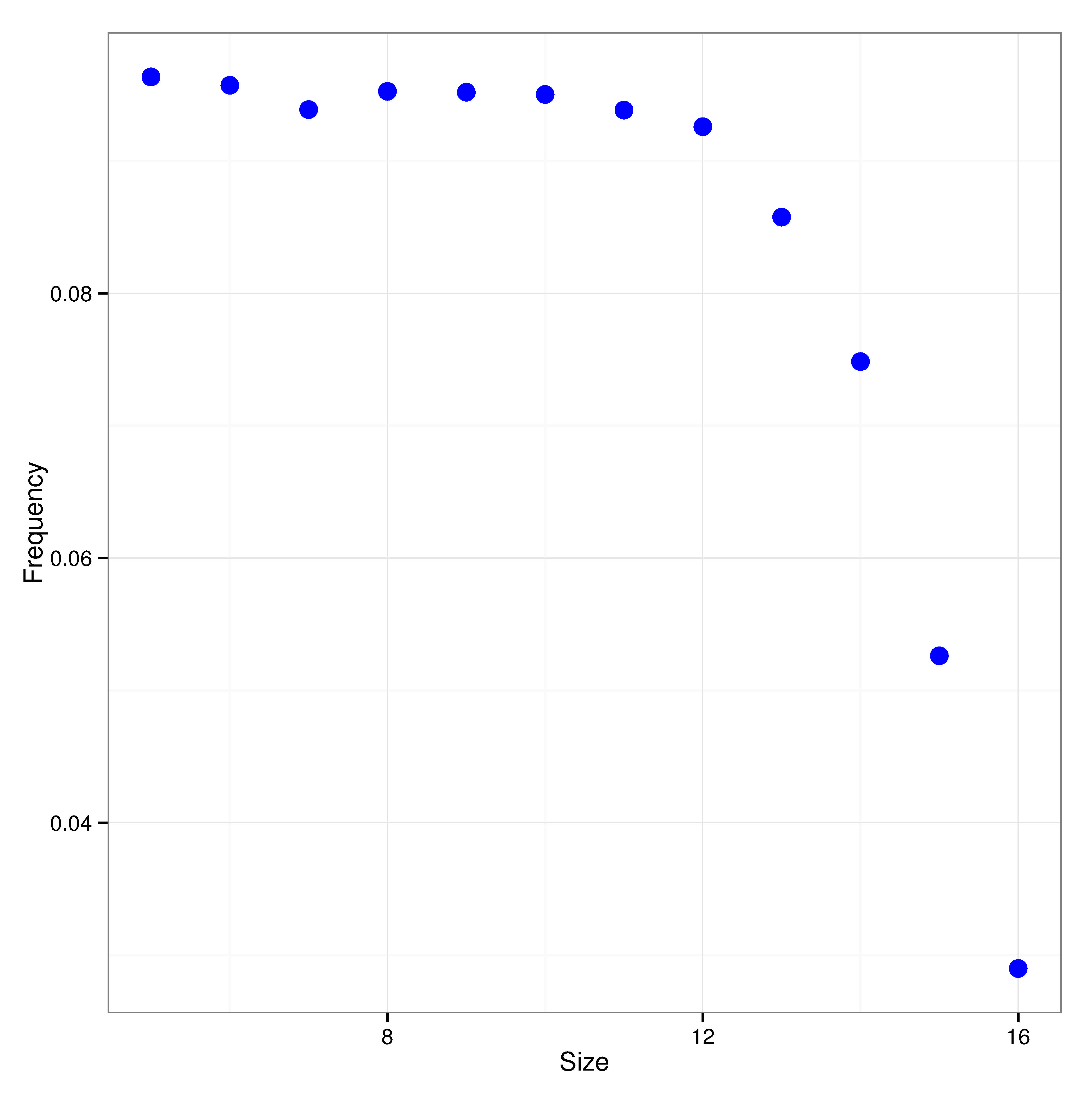

Supplement: S1 Fig — (TIF) [file pone.0148459.s001.tif]

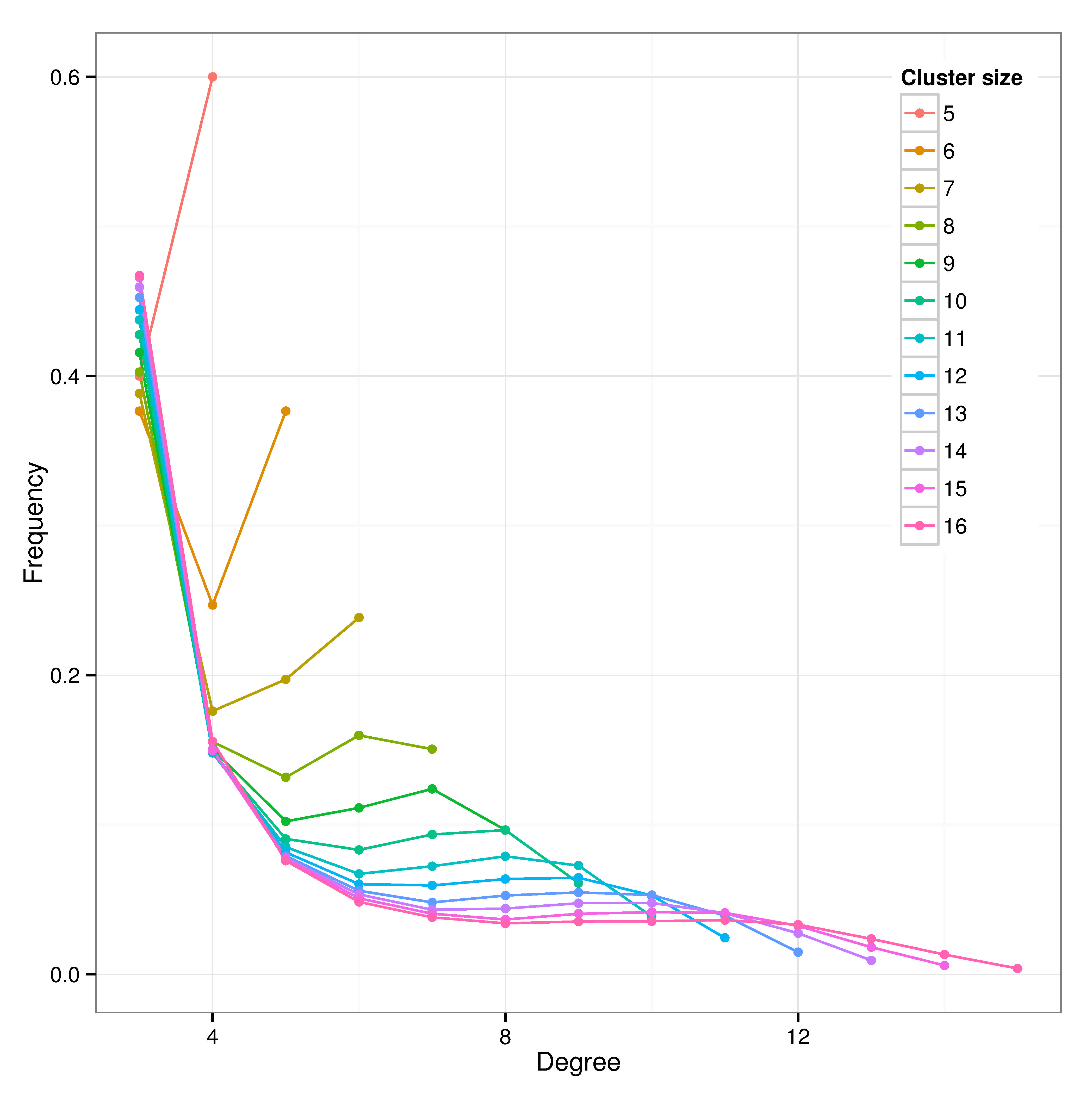

Supplement: S2 Fig — (TIF) [file pone.0148459.s002.tif]
